# OTME-20. Chitinase-3-like-1(CHI3L1) Protein Complexes Regulate the immunosuppressive Microenvironment in Glioblastoma

**DOI:** 10.1093/noajnl/vdab070.071

**Published:** 2021-07-05

**Authors:** Apeng Chen, Yinan Jiang, Zhengwei Li, Xiangwei Xiao, Dean Yimlamai, Ian Pollack, Carlos Camacho, Baoli Hu

**Affiliations:** 1 Department of Neurological Surgery,University of Pittsburgh School of Medicine, Pittsburgh, PA, USA; 2 UPMC Children’s Hospital of Pittsburgh, Pittsburgh, PA, USA; 3 Department of Pediatric Surgery, University of Pittsburgh School of Medicine, Pittsburgh, PA, USA; 4 , Department of Pediatrics, Yale University School of Medicine, New Haven, CT, USA; 5 Department of Computational and Systems Biology, University of Pittsburgh School of Medicine, Pittsburgh, PA, USA

## Abstract

Glioblastoma (GBM) is the most common and highly malignant brain tumor in adults. Despite advances in multimodal treatment, GBM remains largely incurable. While immunotherapies have been highly effective in some types of cancer, the disappointing results from clinical trials for GBM immunotherapy represent continued challenges. GBM is highly immunosuppressive and resistant to immunotherapy because of glioma cells escaping from immune surveillance by reprograming the tumor microenvironment (TME). However, understanding the mechanisms of immune evasion by GBM remains elusive. Based on unbiased approaches, we found that Chitinase-3-like-1 (CHI3L1), also known as human homolog YKL-40, is highly expressed in GBM, which is regulated by the CHI3L1-PI3K/AKT/mTOR signaling in a positive feedback loop. Gain- and loss-function studies reveal that CHI3L1 plays a predominant role in regulating an immunosuppressive microenvironment by reprogramming tumor-associated macrophages (TAMs). Using the liquid chromatography-mass spectrometry and orthogonal structure-based screening, we found that Galectin-3 binding protein (Gal3BP) and its binding partner, Galectin-3 (Gal3), can interact competitively with the same binding motif on CHI3L1, leading to selective migration of M2-like versus M1-like bone marrow-derived macrophages (BMDMs) and resident microglia (MG). Mechanistically, the CHI3L1-Gal3 protein complex governs a transcriptional program of NFκB/CEBPβ to control the protumor phenotype of BMDMs, leading to inhibition of T cell infiltration and activation in the GBM TME. However, Gal3BP can reverse CHI3L1-Gal3 induced signaling pathway activation and subsequent protumor phenotype in TAMs. Based on protein binding motifs, a newly developed Gal3BP mimetic peptide can attenuate immune suppression and tumor progression in the syngeneic GBM mouse models, including decreasing M2-like TAMs and increasing M1-like TAMs and T cell infiltration. Together, these results shed light on the role of CHI3L1 protein complexes in immune evasion by glioblastoma and as a potential immunotherapeutic target for this devastating disease.

